# Mitigating Zoonotic Risks in Intensive Farming: Solutions for a Sustainable Change

**DOI:** 10.1007/s10393-022-01605-8

**Published:** 2022-06-29

**Authors:** Mariëlle Stel, Janina Eggers, Wladimir J. Alonso

**Affiliations:** 1 Department of Psychology of Conflict, Risk, and Safety, University of Twente, De Zul 10, 7522 NJ Enschede, The Netherlands; 2 Welfare Footprint Project, Florianópolis, Santa Catarina Brazil; 3 EPIDOT, Department of Public Health, Federal University of Santa Catarina, Florianópolis, Brazil

Zoonoses—diseases and infections caused by pathogens that are transmitted between animals and humans—are considered one of the most important threats to public health (Bueno-Marí et al. 2015). Seventy-five percent of all emerging human infectious diseases originate from animals (Taylor et al. 2001). These zoonoses have become more frequent and with more consequences for animals and humans (Coker et al. 2011; Wolfe et al. 2007). One of the main routes for the spillover and amplification of pathogens that can affect humans is the production and consumption of animal-based products (Coker et al. 2011; Leibler et al. 2009; Wolfe et al. 2007). The sharp increase in the number of farmed animals over the past 50 years and the higher density at which they are kept, amplified zoonotic risks (Espinoza et al. 2020; Leibler et al. 2009; Silbergeld 2016; Wallace 2016). To mitigate these risks, governmental policies and producer practices have focused on increasing biosecurity (any practice preventing pathogen introduction and release into areas one tries to protect, Leibler et al. 2009) and biocontainment (any measure taken to prevent disease spreading within a herd or flock when the disease is already present, Dargatz et al. 2002). But are these approaches effective?

## Modern Intensive Farming Methods

Theoretically the risk of disease spillover from pathogens circulating in wild animals to farmed animals and the risk of zoonotic disease emergence within intensive farms would be minimized in the environmentally controlled and confined space of the intensive systems (Espinoza et al. 2020*;* Otte et al. 2007). However, below we will argue and show that measures of biosecurity and biocontainment to reduce zoonotic risks are insufficient as (1) diseases are still transmitted between indoor and outdoor environments, even if biosecurity protocols are implemented and (2) intensive farming systems amplify zoonotic risks.

First, pathogens are still transmitted from outdoor environments into intensive farmed animals and vice versa, for instance via personnel, veterinarians, transportation teams, (ventilated) air, (un)treated contaminated water, animal waste, and animal transportation (Graham et al. 2008; Greger 2007a; Otte et al. 2007; Rodríguez-Lázaro et al. 2011; Schuck-Paim and Alonso 2020; Silbergeld 2016). Animal pathogens can also infect humans via the handling and consumption of animals (Newell et al. 2010; Rodríguez-Lázaro et al. 2011). Research shows that although biosecurity, movement restrictions, and prompt isolation of infected farms reduce infectious disease transmission, the risk is far from eradicated (Dhingra et al. 2018; Garske et al. 2007; Schuck-Paim and Alonso 2020). For example, multiple avian influenza outbreaks take place every year in large-scale industrial farms, even though this virus naturally circulates among wild birds (Dhingra et al. 2018; Graham et al. 2008). Many of these outbreaks occur in developed countries where biosecurity protocols are supposedly implemented (Dhingra et al. 2018; Otte et al. 2007). While avian influenza has not yet achieved sustained transmission between humans, a mutation that confers this possibility could easily evolve (Sutton 2018).

Second, intensive farming amplifies the risks and impact of zoonoses compared with small scale farming. The chances that animals develop a disease—either originated within or outside farms—is higher in intensive farming as the conditions lead to immunosuppressed animals. Factors that cause this immunosuppression are, amongst others, intensive selection for fast-growth and productivity (in detriment of other biological features, such as immune function, Van der Most et al. 2011), exposure to high levels of ammonia and fecal dust (Greger 2007b), animals’ genetic homogeneity (Springbett et al. 2003), and the highly stressful physical and psychological conditions animals live and are transported in (El-Lethey et al. 2003; Rostagno 2009). Research indeed shows that the odds of avian influenza outbreaks are higher in large-scale farms than in smaller backyard flocks (Dhingra et al. 2018). Moreover, high animal density leads to rapid pathogen spread within the farm (Dhingra et al. 2018) and, together with lack of genetic diversity, it increases the risk for pathogens to mutate into zoonoses (Espinoza et al. 2020). Evidence shows that conversions from low into high pathogenic viruses—which leads to the emergence of zoonoses—mostly occurred in high density locations (Graham et al. 2008).

## Toward a Solution

Taken together, we conclude that modern intensive farming is not part of the solution, but poses a severe risk for the emergence of zoonoses, including the ones with pandemic potential. Policy measures to increase biocontainment and biosecurity are far from sufficient to mitigate such risks. As the emergence of zoonoses increased alongside increased demand for animals and their products (Coker et al. 2011; Espinoza et al. 2020*,* Karesh et al. 2012; Leibler et al. 2009), a solution that reduces the risks posed by animal farming is critically needed. Extensively farmed animals present overall less epidemiological risks (Dhingra et al. 2018; Graham et al. 2008), but they are less capable of feeding an increasing global demand for affordable, convenient, and tasteful source of proteins. Hence, from a public health perspective, a protein shift toward substitutes that are being increasingly made available through breakthroughs in food technology, such as plant-based and cultured meat options, offers a viable solution to mitigating zoonotic risks (e.g., Hong et al. 2021; Santo et al. 2020; Shapiro 2018). This protein transition also reduces CO2 emissions, environmental pollution, and natural habitat destruction and eliminates farmed animals’ suffering, and contributes to people’s health and food security (e.g., Kona-Boun 2020; Shapiro 2018; Song et al. 2016; Springmann et al. 2016; Sun et al. 2022).

Despite all those positive advantages, only a few countries have so far demonstrated their active commitment to the protein transition. One of the few examples is the Danishs’ government who spend 1.25 billion Kroner (191,5 million Dollars) to subsidize the development and promotion of plant-based products. Also, they presented consumption guidelines for citizens which include the advice to consume less meat and choose plant-based consumption. This already reduced Danishs’ meat consumption (Bakker 2021). An example of reluctance toward this transition is the Dutch government removing the advice to eat less meat from their climate awareness campaign as the advice would be too sensitive (NLTimes 2021). People, however, are open to the protein transition (e.g., Epson 2021; Van Heck et al. 2022) and feel positive when they make sustainable food choices (Zawadzki et al. 2020). Nevertheless, they experience barriers to change their behavior and express a responsibility for governments to reduce zoonotic risks (e.g., Stel et al. 2022).

## What Governments Can Do

Epidemiologists advised, among others, to reduce the number of animals held and to facilitate a transition to products that do not involve their rearing and butchering (Bekedam et al. 2021). Importantly, given the direct public health problems and externalities involved in the production of conventional animal meat, dairy, and eggs, governments should stop subsidizing this industry and start subsidizing and stimulating protein alternatives instead (Brozek and Falkenberg 2021; Schuck-Paim 2020; Wiebers and Feigin 2020).

Alternative protein consumption can be stimulated by lowering the price of high protein fruit, vegetables, and alternatives for animal products (e.g., tax-free or taxing based on sustainability) as one barrier to plant-based consumption is that people desire affordable products (Globescan 2020; Thøgersen 2021). Also, the availability, visibility, and normativity of plant-based and cultured meat options should be increased to stimulate the protein transition (see Cialdini and Jacobson 2021; Pechey et al. 2022). Another barrier to plant-based consumption is people’s belief that one’s contribution to the solution is minimal. Therefore, the link between purchases and their impact on sustainability needs to be made clear (Meijers et al. 2021; Thøgersen 2021). This can be done by adding the “animal welfare footprint” (Alonso and Schuck-Paim 2021) on food packages and receipts, indicating animals’ quality of life. This footprint is to a great extent a proxy of zoonotic risks (e.g., density, stress, immune function; Scherer et al. 2018).

Another advice for governments to reduce zoonotic risks is to inform people about zoonoses (Bekedam et al. 2021). People should be informed about the probability and the severity of zoonotic risks and about which behavioral changes are recommended as these factors lead to behavior change (Floyd et al. 2000; Ruiter et al. 2014). An intervention message including these factors showed more positive attitudes toward behaviors reducing zoonotic risks (Stel and Banach 2022). Also, when aware that intensive farming plays a role in zoonotic risks, a majority agrees to lower their animal product consumption (Stel et al. 2022).

To conclude, the protein transition is urgent due to zoonotic, environmental, animal welfare, and people’s health issues, and is supported by a majority of people when they are informed of these externalities (Epson 2021; Stel et al. 2022; Van Heck et al. 2022). Governments should encourage alternatives to slaughtered meat, animal dairy, and eggs by (1) removing the direct and indirect system of subsidies to the animal industry, (2) subsidizing the development and promotion of plant-based and cultured meat products, and (3) providing information on zoonotic disease risk, including (a) removing barriers that prevent the public and scientific scrutiny of the externalities in these production systems and (b) making the link between animal product consumption and its effect on personal and global health clear.
